# A publishing infrastructure for Artificial Intelligence (AI)-assisted academic authoring

**DOI:** 10.1093/jamia/ocae139

**Published:** 2024-06-14

**Authors:** Milton Pividori, Casey S Greene

**Affiliations:** Department of Biomedical Informatics, University of Colorado School of Medicine, Aurora, CO 80045, United States; Department of Genetics, Perelman School of Medicine, University of Pennsylvania, Philadelphia, PA 19104, United States; Department of Biomedical Informatics, University of Colorado School of Medicine, Aurora, CO 80045, United States; Center for Health AI, Department of Biomedical Informatics, University of Colorado School of Medicine, Aurora, CO 80045, United States

**Keywords:** artificial intelligence, large language models, scholarly publishing, Manubot

## Abstract

**Objective:**

Investigate the use of advanced natural language processing models to streamline the time-consuming process of writing and revising scholarly manuscripts.

**Materials and Methods:**

For this purpose, we integrate large language models into the Manubot publishing ecosystem to suggest revisions for scholarly texts. Our AI-based revision workflow employs a prompt generator that incorporates manuscript metadata into templates, generating section-specific instructions for the language model. The model then generates revised versions of each paragraph for human authors to review. We evaluated this methodology through 5 case studies of existing manuscripts, including the revision of this manuscript.

**Results:**

Our results indicate that these models, despite some limitations, can grasp complex academic concepts and enhance text quality. All changes to the manuscript are tracked using a version control system, ensuring transparency in distinguishing between human- and machine-generated text.

**Conclusions:**

Given the significant time researchers invest in crafting prose, incorporating large language models into the scholarly writing process can significantly improve the type of knowledge work performed by academics. Our approach also enables scholars to concentrate on critical aspects of their work, such as the novelty of their ideas, while automating tedious tasks like adhering to specific writing styles. Although the use of AI-assisted tools in scientific authoring is controversial, our approach, which focuses on revising human-written text and provides change-tracking transparency, can mitigate concerns regarding AI’s role in scientific writing.

## Introduction and background

Scholarly writing has evolved since the first scientific journals 350 years ago, adopting practices like external peer review in the last century.^1^^,^^2^ It often involves dense language to convey new advances or literature summaries.^3^ Meanwhile, recent computing advances have enabled large language models (LLMs) like OpenAI’s GPT-3 and GPT-4,^4^ revolutionizing technologies and applications in various fields, including medical informatics and scientific communication.^5^^,^^6^ These models promise to streamline scientific writing,^7^ though their use raises accuracy and ethical concerns.^8^^,^^9^

We introduce a human-centric AI method for scholarly writing, leveraging LLMs for draft revision within the Manubot platform, a tool for collaborative publishing.^10^ Here, we propose the Manubot AI Editor, which suggests revisions via GitHub, balancing AI’s efficiency with human oversight to ensure accuracy. Tested on 5 manuscripts, we found it maintained the original meaning, improved style, and handled complex expressions, proving a valuable addition to the Manubot suite. We anticipate our tool will help authors more effectively communicate their work.

## Objective

This study has 2 objectives: (1) investigate how recently released LLMs can reduce the time-consuming process of writing and revising scholarly manuscripts; (2) develop an approach for incorporating these models into the Manubot publishing ecosystem to transparently suggest revisions to improve the quality of the original text.

## Materials and methods

We propose a human-centric approach for the use of AI in manuscript writing, which consists of the following steps: (1) human authors write the manuscript content; (2) an LLM revises the manuscript, generating a set of suggested changes; (3) human authors review the suggested changes, and the approved edits are then integrated into the manuscript. By focusing on human review, this approach attempts to mitigate the risk of generating incorrect or misleading information. To implement this human-centric approach, we developed a tool called the Manubot AI Editor, which is part of the Manubot infrastructure for scholarly publishing.^10^

### Overview of the Manubot AI editor

**Figure 1.**
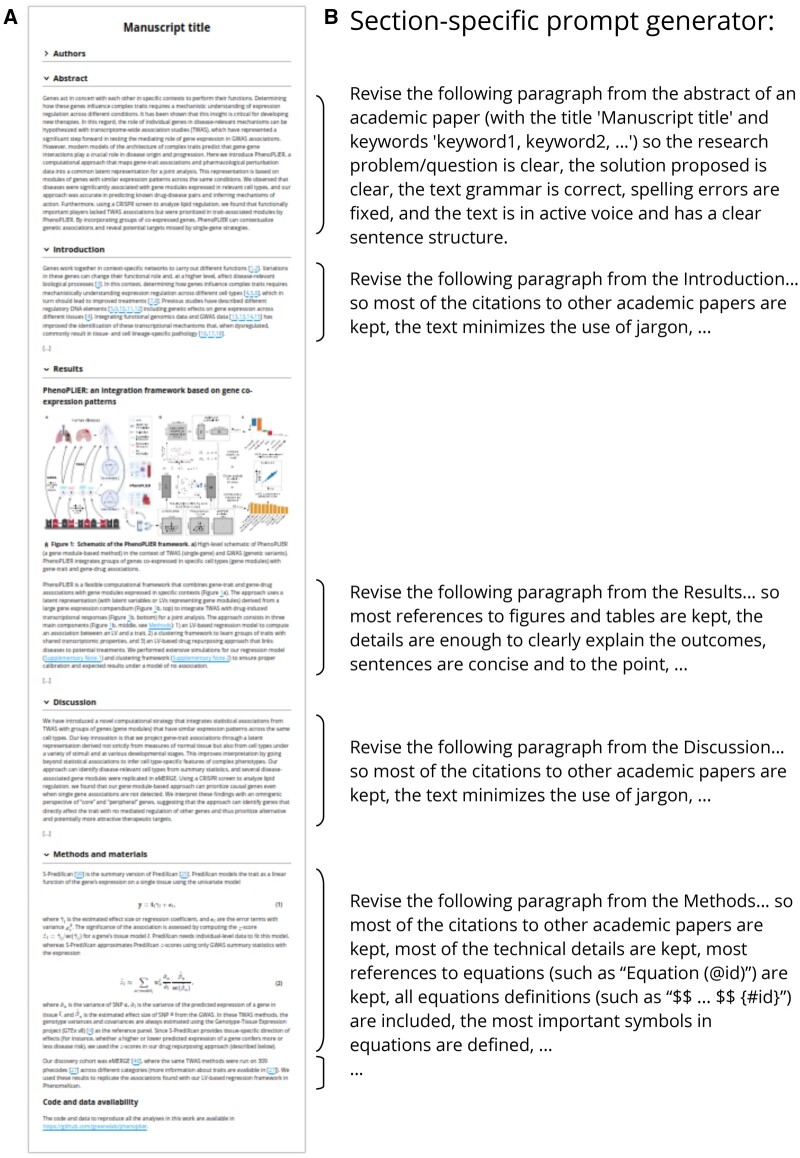
AI-based revision applied on a Manubot-based manuscript. (A) A manuscript written with Manubot containing different sections (image based on preprint at https://greenelab.github.io/manubot-gpt-manuscript/). (B) The prompt generator integrates metadata using prompt templates to generate section-specific prompts for each paragraph. If a paragraph belongs to a non-standard section, then a default prompt will be used to perform a basic revision only. The prompt for the “Methods” section includes the formatting of equations with identifiers. All sections’ prompts include these instructions: “the text grammar is correct, spelling errors are fixed, and the text has a clear sentence structure,” although these are only shown for abstracts. Our tool allows the user to provide a custom prompt instead of using the default ones shown here.

The Manubot AI Editor is an AI-based revision infrastructure integrated into Manubot,^10^ a tool for collaborative writing of scientific manuscripts. Manubot integrates with popular version control platforms such as GitHub, allowing authors to easily track changes and collaborate on writing in real time. Furthermore, Manubot automates the process of generating a formatted manuscript (eg, HTML, PDF, DOCX; Figure 1A shows the HTML output). Built upon this modern and open paradigm, our Manubot AI Editor (https://github.com/manubot/manubot-ai-editor) includes 3 components: (1) a Python library that provides classes and functions to read the manuscript content and its metadata, calls the LLM for automatic text revision, and writes the results back; (2) a GitHub Actions workflow that uses our Python library within GitHub to preserve provenance information for transparency; (3) a prompt generator that integrates the manuscript’s metadata using prompt templates to generate section-specific prompts for each paragraph (Figure 1B).

The GitHub Actions workflow enables users to easily trigger an automated revision task on either the entire manuscript or specific sections of it. When the action is triggered, the manuscript is parsed by section and then by paragraph (Figure 1B), which are then passed to the language model along with a set of custom prompts. The model subsequently returns a revised version of the text. Our workflow leverages the GitHub API to generate a new pull request (PR), allowing the user to review and modify the output before merging the changes into the manuscript. This workflow assigns text attribution to either the human user or the AI language model, which may be important in light of potential future legal decisions that could reshape the copyright landscape concerning the outputs of generative models.

We used the OpenAI API for access to these models. Since this API incurs a cost with each run that depends on the manuscript length, we implemented a workflow in GitHub Actions that can be manually triggered by the user. Our implementation allows users to tune the costs to their needs by enabling them to select specific sections for revision instead of the entire manuscript. Additionally, several model parameters can be adjusted to further tune costs, such as the language model version (including the current GPT-3.5 Turbo and GPT-4, and potentially newly published ones), how much risk the model will take, or the “quality” of the completions. For instance, using Davinci models, the cost per run is under $0.50 for most manuscripts. More details about the implementation, installation, and usage of the Manubot AI Editor can be found in the Supplementary Material.

## Results

### Evaluation setup

We used 5 different manuscripts for the evaluation of our AI-based revision workflow (see below), and during the prompt engineering phase (see below), we also used a unit testing framework to ensure that the revisions produced by our prompts met a minimum set of quality measures (see Supplementary Material).

We evaluated our AI-assisted revision workflow using 2 models from OpenAI: Davinci (text-davinci-003) and GPT-3.5 Turbo (gpt-3.5-turbo). The first one is based on GPT-3 Davinci models and used to be a production-ready model, although it was now succeeded by the new GPT-3.5 Turbo and GPT-4 models. We used the most capable GPT-4 Turbo model for evaluating the revisions (LLM-as-a-Judge).

#### Manuscripts

For the evaluation of our tool, we conducted manual assessments performed by humans and automatic assessments performed by an LLM. For the human assessments, we used 3 of our own manuscripts (Table 1): the Clustermatch Correlation Coefficient (CCC),^11^ PhenoPLIER,^12^ and Manubot-AI (this manuscript). CCC is a new correlation coefficient applied to transcriptomic data, while PhenoPLIER is a framework consisting of 3 different methods used in genetic studies. CCC falls under the field of computational biology, whereas PhenoPLIER pertains to genomic medicine. CCC outlines 1 computational method applied to a specific data type (correlation to gene expression). In contrast, PhenoPLIER describes a framework that integrates 3 different approaches (regression, clustering, and drug-disease prediction) using data from genome-wide association studies and transcription-wide association studies (GWAS and TWAS), gene expression, and transcriptional responses to small molecule perturbations. Thus, CCC has a simpler structure, while PhenoPLIER is a more complex manuscript with additional figures and tables, along with a “Methods” section that includes equations. The third manuscript, Manubot-AI, has a much simpler structure and was written and revised using our tool prior to submission, demonstrating a practical AI-based revision use case. For the automatic assessments, we incorporated 2 external manuscripts (with IDs BioChatter and Epistasis in Table 1).

**Table 1.** Manuscripts used to evaluate the AI-based revision workflow.

Manuscript ID	GitHub URL	Title	Keywords
CCC	greenelab/ccc-manuscript	An efficient not-only-linear correlation coefficient based on machine learning	Correlation coefficient, nonlinear relationships, gene expression
PhenoPLIER	greenelab/phenoplier_manuscript	Projecting genetic associations through gene expression patterns highlights disease etiology and drug mechanisms	Genetic studies, functional genomics, gene co-expression, therapeutic targets, drug repurposing, clustering of complex traits
Manubot-AI	greenelab/manubot-gpt-manuscript	A publishing infrastructure for AI-assisted academic authoring	Manubot, artificial intelligence, scholarly publishing, software
Epistasis	quinlan-lab/mutator-epistasis-manuscript	Epistasis between mutator alleles contributes to germline mutation rate variability in laboratory mice	
BioChatter	biocypher/biochatter-paper	A Platform for the Biomedical Application of LLMs	Biomedicine, LLMs, framework, retrieval-augmented generation, knowledge graph

The title and keywords of a manuscript are used in prompts for revising paragraphs. IDs are used in the text to refer to them.

#### Evaluation using human assessments

We enabled the Manubot AI revision workflow in the GitHub repositories of the 3 manuscripts (CCC, PhenoPLIER, and Manubot-AI). This added the “ai-revision” workflow to the “Actions” tab of each repository. We manually triggered the workflow and utilized the text-davinci-003 language model to generate 1 PR per manuscript. These PRs can be accessed from the “Pull requests” tab of each repository. The PRs display all the differences between the original text and the AI-based revision suggestions.

When manually assessing the quality of the revisions, we considered whether the revision: (1) preserve the original meaning, (2) preserve important details, (3) introduced new and incorrect information, and (4) preserve the correct Markdown format (eg, citations, equations).

#### Evaluation using an LLM as a judge

For this evaluation, we ran our workflow on manuscripts CCC, PhenoPLIER, BioChatter, and Epistasis using the GPT-3.5 Turbo model (gpt-3.5-turbo). We then inspected each PR and manually matched all pairs of original and revised paragraphs, across the abstract, introduction, methods, results, and Supplementary Material sections. This procedure generated 31 paragraph pairs for CCC, 63 for PhenoPLIER, 37 for BioChatter, and 63 for Epistasis. Using the LLM-as-a-Judge method,^13^ we evaluated the quality of the revisions using both GPT-3.5 Turbo (gpt-3.5-turbo) and GPT-4 Turbo (gpt-4-turbo-preview) as judges. The judge is asked to decide which of the 2 paragraphs in each pair is better or if they are equally good (tie). For this, we used prompt chaining, where the judge first evaluates the quality of each paragraph independently by writing a list with positive and negative aspects in the following areas: (1) clear sentence structure, (2) ease of understanding, (3) grammatical correctness, (4) absence of spelling errors. Then, the judge was asked to be as objective as possible and decide if 1 of the paragraphs is clearly better than the other or if they are similar in quality, while also providing a rationale for the decision. We also accounted for the case of position bias^13^ (ie, the order in which the paragraphs were presented could influence the decision) by swapping the order of the paragraphs. Each assessment was repeated 10 times. The full prompt chain can be seen in File S4, which includes an example of the output in each step generated by GPT-4 Turbo as a judge.

### Human assessments across different sections

Following our criteria for human assessments (see above), we inspected the PRs generated by the AI-based workflow and reported on our assessment of the changes suggested by the tool across different sections of the manuscripts. The reader can access the PRs in the manuscripts’ GitHub repositories (Table 1) and also included as diff files in File S1 (CCC), File S2 (PhenoPLIER), and File S3 (Manubot-AI).

Below, we present the differences between the original text and the revisions made by the tool in a diff format (obtained from GitHub). Line numbers are included to show the length differences. Unless the AI suggestions represent a complete overhaul of the text, single words are underlined and highlighted in colors to more clearly see the differences within a single sentence. Red indicates words removed by the tool, while green indicates words added; no underlining indicates words kept unchanged. In the GitHub repositories, the full diffs can be seen by clicking on the “Files changed” tab under each PR.

#### Abstract

**Figure 2.**
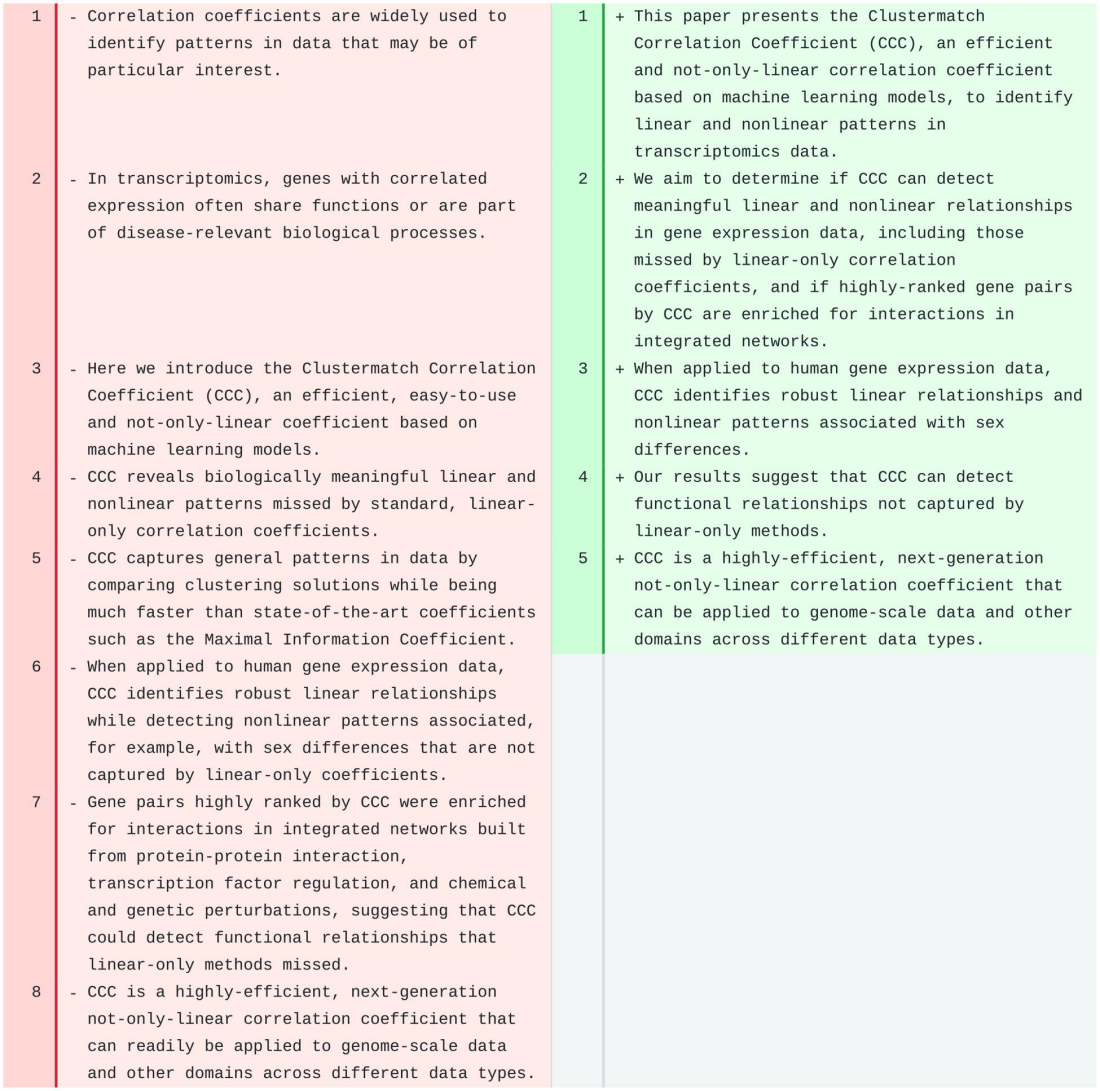
Abstract of CCC. Original text is on the left and suggested revision on the right. Single words are not underlined/highlighted in this case because the revision completely overhauled the text.

We applied the AI-based revision workflow to the CCC abstract (Figure 2). The tool completely rewrote the text, leaving only the last sentence mostly unchanged. The text was significantly shortened, and the sentences were longer than those in the original, which could make the abstract slightly harder to read. The revision removed the first 2 sentences, which introduced correlation analyses and transcriptomics, and directly stated the purpose of the manuscript. It also removed details about the method (line 5) and focused on the aims and results obtained, ending with the same last sentence, suggesting a broader application of the coefficient to other data domains (as originally intended by the authors of CCC). The main concepts were still present in the revised text.

The revised text for the abstract of PhenoPLIER was significantly shortened (from 10 sentences in the original, to only 3 in the revised version). However, in this case, important concepts (such as GWAS, TWAS, CRISPR) and a proper amount of background information were missing, producing a less informative abstract.

#### Introduction

**Figure 3.**
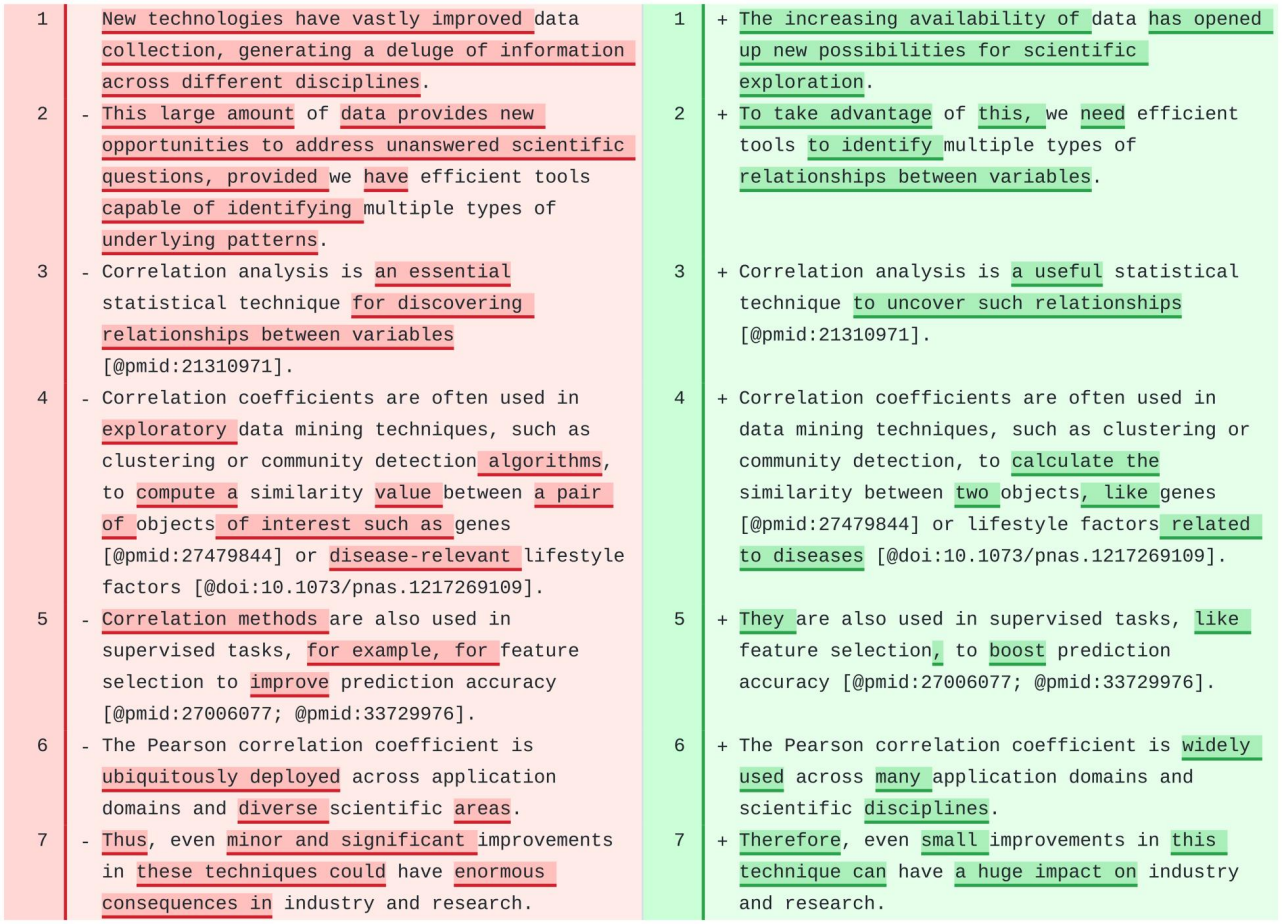
First paragraph in the “Introduction” section of CCC. Original text is on the left and suggested revision on the right.

The tool significantly revised the “Introduction” section of CCC (Figure 3), producing a more concise and clear introductory paragraph. The revised first sentence concisely incorporated ideas from the original 2 sentences, introducing the concept of “large datasets” and the opportunities for scientific exploration. The model generated a more concise second sentence introducing the “need for efficient tools” to find “multiple relationships” in these datasets. The third sentence connected nicely with the previous one. All references to scientific literature were kept in the correct Manubot format, even though our prompts do not specify the references format. The rest of the sentences in this section were also correctly revised and could be incorporated into the manuscript with minor or no further changes.

We also observed a high-quality revision of the introduction of PhenoPLIER. However, the model failed to maintain the format of citations in 1 paragraph. Additionally, the model did not converge to a revised text for the last paragraph, and our tool left an error message as an HTML comment at the top: The AI model returned an empty string. Debugging the prompts revealed this issue, which could be related to the complexity of the paragraph. In these cases, rerunning the automated revision might solve this type of issue.

#### Results

**Figure 4.**
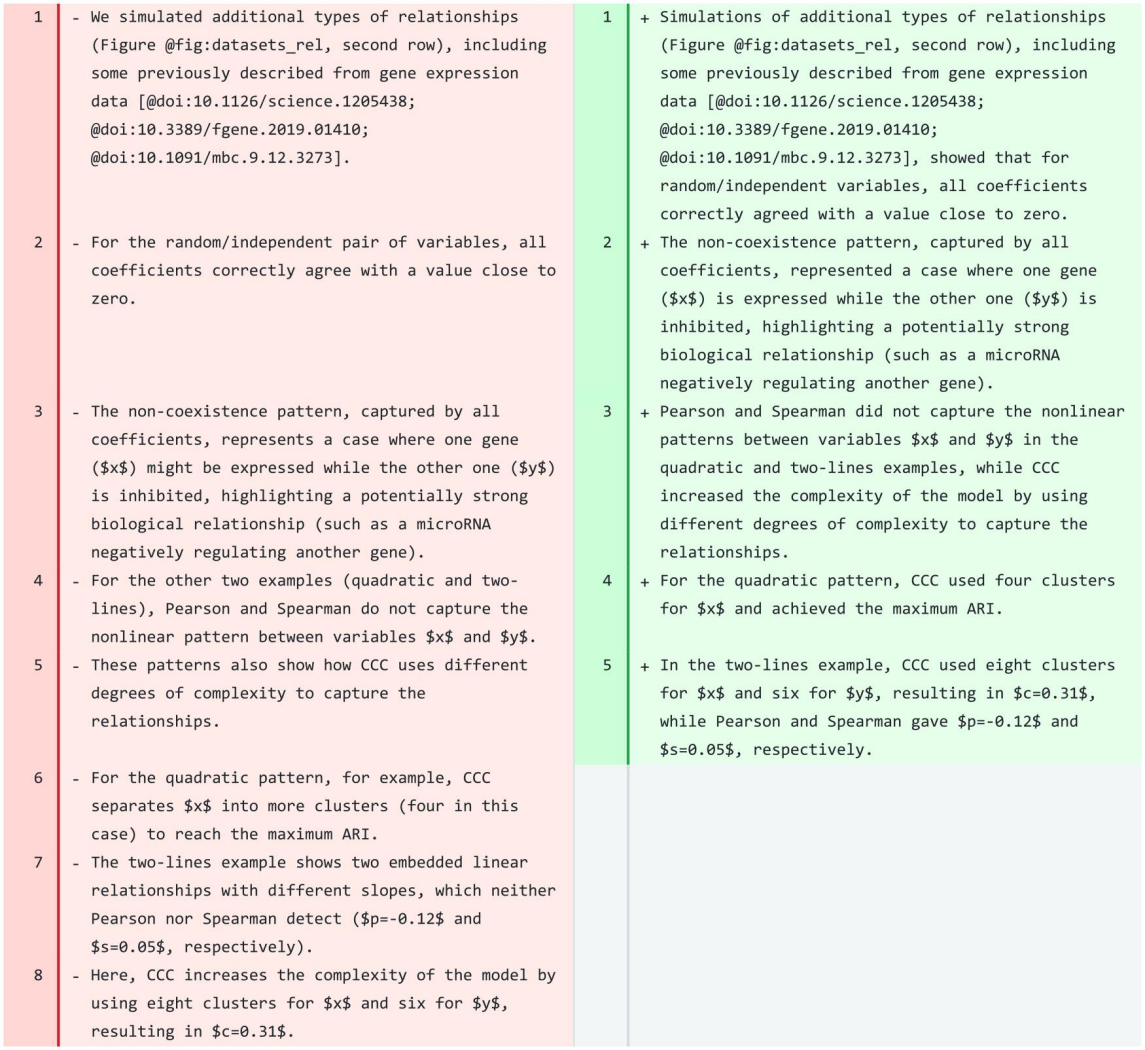
A paragraph in the “Results” section of CCC. Original text is on the left and suggested revision on the right. Single words are not underlined/highlighted in this case because the revision completely overhauled the text.

We tested the tool on a paragraph from the “Results” section of CCC (Figure 4). This paragraph describes Figure 1 of the CCC manuscript,^11^ which showcases 4 different datasets, each with 2 variables, and various relationships or patterns labeled as random/independent, non-coexistence, quadratic, and two-lines. The revised paragraph, while having fewer sentences, is slightly longer and consistently uses the past tense, unlike the original one which has tense shifts. The revised paragraph also retains all citations, which, although not explicitly mentioned in the prompts for this section (as it is for introductions), is important in this case. The original LaTeX format was maintained for the math, and the figure was correctly referenced using the Manubot syntax. In the third sentence of the revised paragraph (line 3), the model generated a good summary of how all coefficients performed in the last 2 nonlinear patterns, and why CCC was able to capture them. As human authors, we would make a single change at the end of this sentence to avoid repeating the word “complexity”: “…, while CCC increased the model’s complexity to capture the relationships.” The revised paragraph is more concise and clearly describes what the figure shows and how CCC works. It is remarkable that the model rewrote some of the concepts in the original paragraph (lines 4-8) into 3 new sentences (lines 3-5) with the same meaning, but more concisely and clearly. The model also produced high-quality revisions for several other paragraphs that would only need minor changes.

However, other paragraphs in CCC required extensive changes before they could be incorporated into the manuscript. For instance, the model generated revised text for certain paragraphs that was more concise, direct, and clear. However, this often resulted in the removal of important details and occasionally altered the intended meaning of sentences. To address this issue, we could accept the simplified sentence structure proposed by the model but reintroduce the missing details for clarity and completeness.

When applied to the PhenoPLIER manuscript, the model produced high-quality revisions for most paragraphs while preserving citations and references to figures, tables, and other sections of the manuscript in the Manubot/Markdown format. In some cases, important details were missing, but they could be easily added back while preserving the improved sentence structure of the revised version. In other cases, the model’s output demonstrated the limitations of revising 1 paragraph at a time without considering the rest of the text. For instance, 1 paragraph described our CRISPR screening approach to assess whether top genes in a latent variable could represent good therapeutic targets. The model generated a paragraph with a completely different meaning (Figure S1). It omitted the CRISPR screen and the gene symbols associated with the regulation of lipids, which were key elements in the original text. Instead, the new text describes an experiment that does not exist, with a reference to a non-existent section. This suggests that the model focused on the title and keywords of the manuscript (Table 1) that were part of every prompt (Figure 1). For example, it included the idea of “gene co-expression” analysis (a keyword) to identify “therapeutic targets” (another keyword) and replaced the mention of “sets of genes” in the original text with “clusters of genes” (closer to the keyword including “clustering”). This was a poor model-based revision, indicating that the original paragraph might be too short or disconnected from the rest and could be merged with the next one, which describes follow-up and related experiments.

#### Discussion

In both the CCC and PhenoPLIER manuscripts, revisions to the “Discussion” section appeared to be of high quality. The model kept the correct format when necessary (eg, using italics for gene symbols), maintained most of the citations, and improved the readability of the text in general. Revisions for some paragraphs introduced minor mistakes that a human author could readily fix.

**Figure 5.**
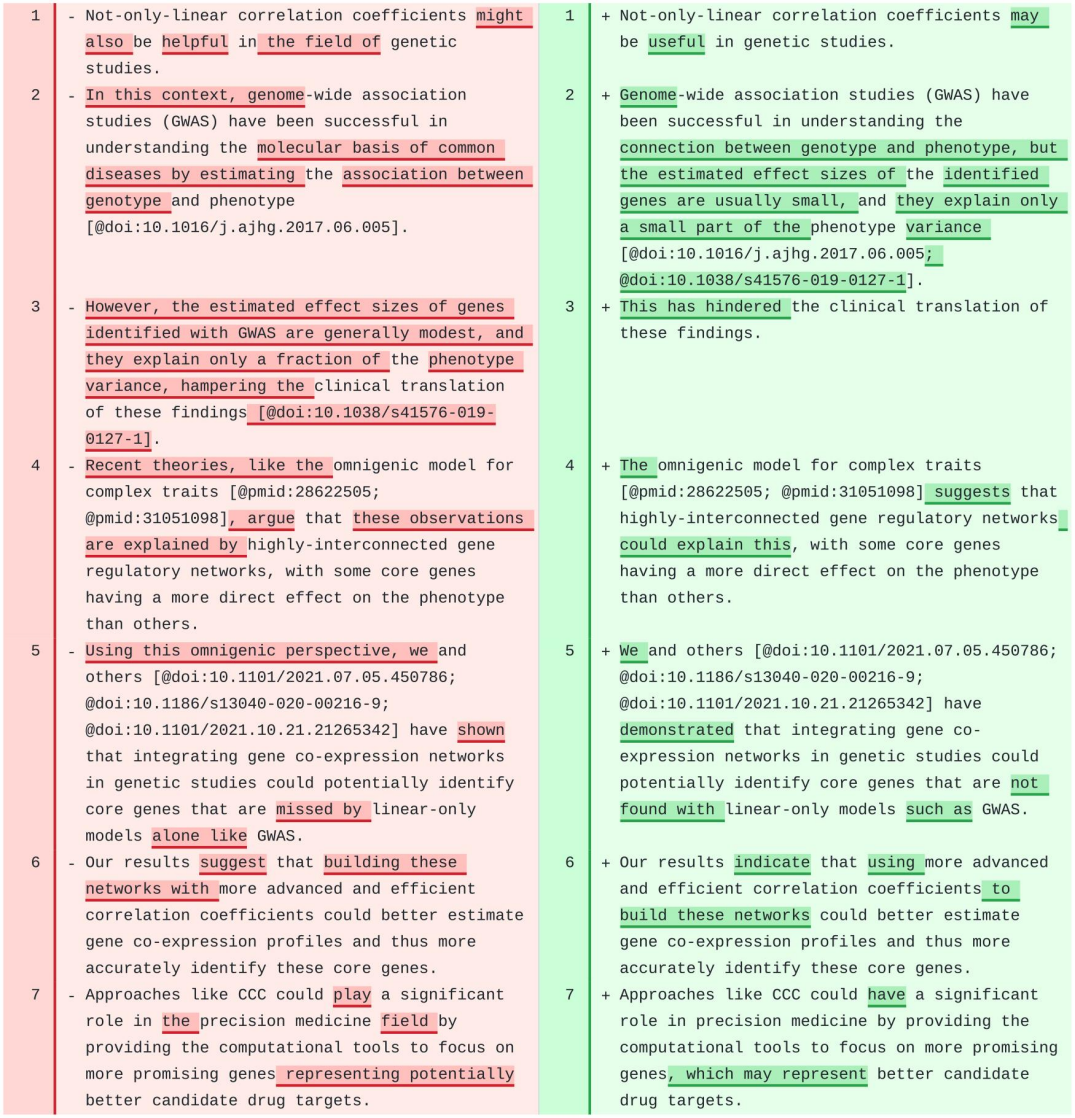
A paragraph in the “Discussion” section of CCC. Original text is on the left and suggested revision on the right.

One paragraph from CCC discusses how not-only-linear correlation coefficients could potentially impact genetic studies of complex traits (Figure 5). Although some minor changes could be incorporated, we believe the revised text reads better than the original. It is also interesting to see how the model understood the format of citations and built more complex structures from it. For instance, the 2 articles referenced in lines 2 and 3 of the original text were correctly merged into a single citation block and separated with a “;” in line 2 of the revised text.

#### Methods

Prompts for the “Methods” section were the most challenging to design, especially when the sections included equations. The prompt for Methods (Figure 1) is more focused in keeping the technical details, which was especially important for PhenoPLIER, whose “Methods” section contains paragraphs with several mathematical expressions.

**Figure 6.**
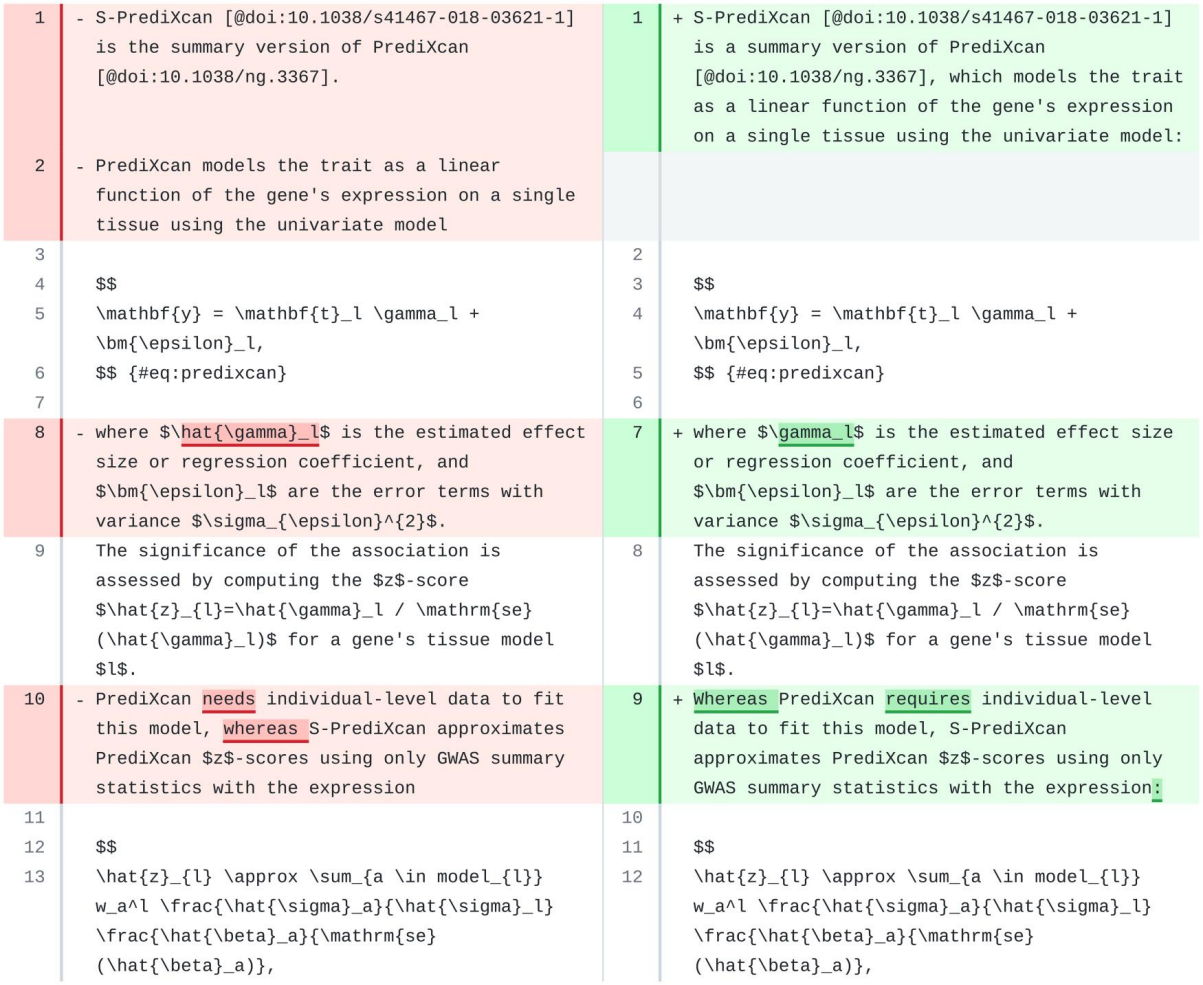
A paragraph in the “Methods” section of PhenoPLIER. Original text is on the left and suggested revision on the right.

We revised a paragraph in PhenoPLIER that contained 2 numbered equations (Figure 6). The model made very few changes, and all the equations, citations, and most of the original text were preserved. However, we found it remarkable how the model identified an incorrect reference to a mathematical symbol (line 8) and corrected it in the revision (line 7). Indeed, the equation with the univariate model used by PrediXcan (lines 4-6 in the original) includes the *true* effect size *γ_l_* (\gamma_l) instead of the *estimated* one ${\hat{\gamma }}_{l}$ (\hat{\gamma}_l).

In PhenoPLIER, we found 1 large paragraph with several equations that the model failed to revise, although it performed relatively well in revising the rest of the section. In CCC, the revision of this section was good overall, with some minor and easy-to-fix issues as in the other sections.

We also observed issues arising from revising 1 paragraph at a time without context. For instance, in PhenoPLIER, 1 of the first paragraphs mentions the linear models used by S-PrediXcan and S-MultiXcan without providing any equations or details. These were presented in the following paragraphs, but since the model had not yet encountered that information, it opted to add those equations immediately (in the correct Manubot/Markdown format).

When revising the “Methods” sections of Manubot-AI (this manuscript), the model, in some cases, added novel sentences containing incorrect information. For example, for 1 paragraph, it included a formula (using the correct Manubot format) presumably to predict the cost of a revision run. In another paragraph (Figure S2), it introduced new sentences stating that the model was “trained on a corpus of scientific papers from the same field as the manuscript” and that its suggested revisions resulted in a “modified version of the manuscript that is ready for submission.” Although these are important future directions, neither statement accurately describes the present work.

### Automated assessments

**Figure 7.**
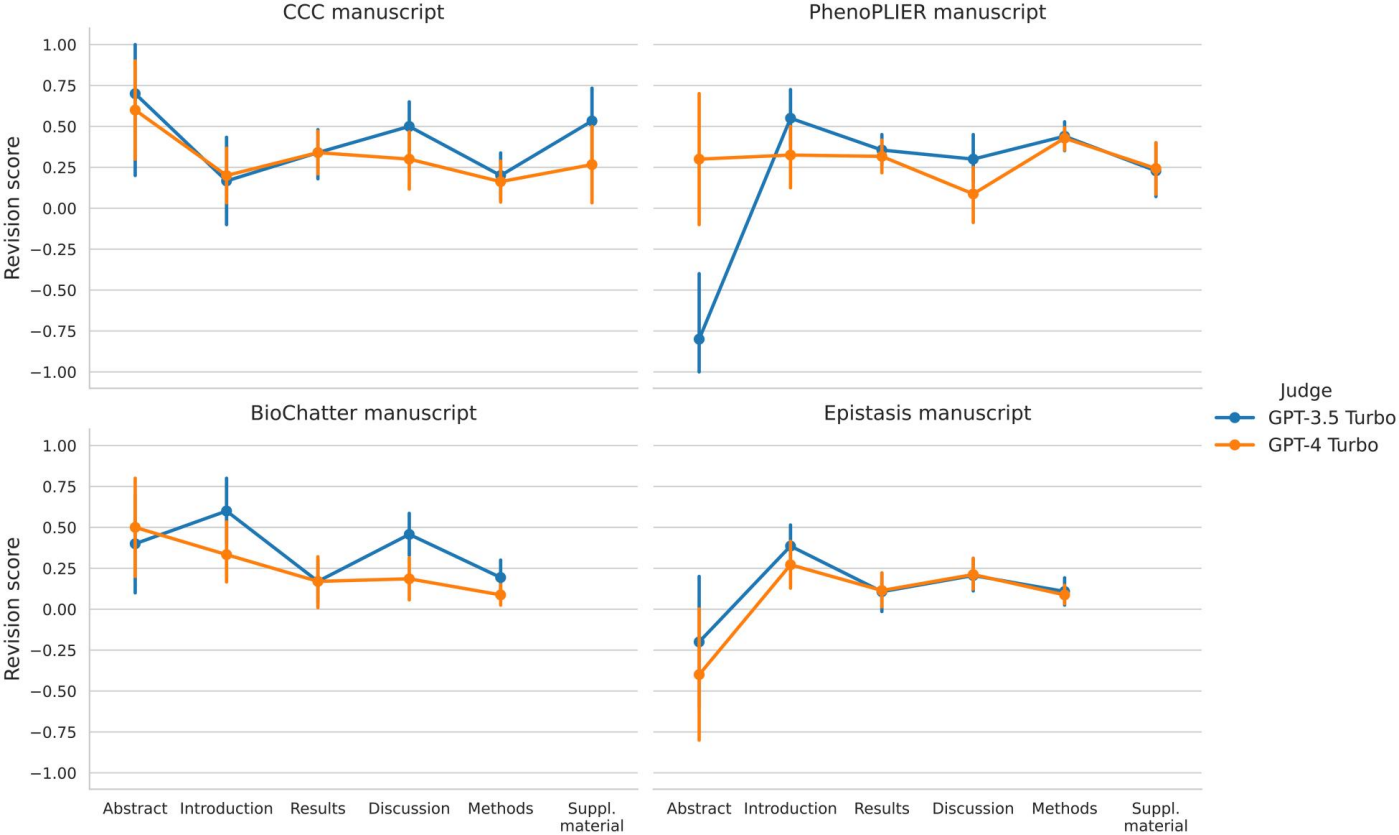
Automated assessment of preference over revised paragraphs. A revision score (*y*-axis) close to 1 indicates that the LLM acting as a judge preferred the revised paragraph over the original one, while a score of −1 indicates the opposite; a score close to zero indicates either a tie or position bias. Each point represents the average score of paragraphs from a section in 1 of the 4 manuscripts: CCC, PhenoPLIER, BioChatter, and Epistasis.

The automatic assessment of paragraphs from different sections across 4 manuscripts is depicted in Figure 7. A revision score above zero indicates that the LLM acting as a judge preferred the revised paragraph over the original one on average, while a score below zero indicates the opposite. It can be seen that the 2 models used as judges, GPT-3.5 Turbo and GPT-4 Turbo, generally agreed and favored the revised paragraphs over the original ones (revision score above zero) in most cases. The only section where the original paragraphs were clearly preferred was the Abstract of the PhenoPLIER and Epistasis manuscripts. GPT-3.5 Turbo showed a preference for the original abstract of PhenoPLIER in most cases, and the model rationale (File S5) aligns with our human assessment: the original abstract provides a more “detailed explanation” of the approaches and a “comprehensive overview of the research.”

## Discussion

Our tool, the Manubot AI Editor, integrates AI-based revision models into the Manubot publishing platform. Writing academic papers can be time-consuming and challenging to comprehend, so we aimed to use technology to assist researchers in communicating their findings more effectively. Our AI-based revision workflow uses a prompt generator that creates manuscript- and section-specific instructions for the language model. Authors can easily trigger this workflow from the GitHub repository to suggest revisions that can be reviewed later. This workflow utilizes OpenAI models, generating a PR of revisions for authors to review. We have established default parameters for these models that perform well for our use cases across different sections and manuscripts. Users also have the option to customize the revision process by selecting specific sections, adjusting the model’s behavior to suit their needs and budget, and even providing custom prompts instead of using the default, section-specific ones. This can be particularly beneficial for specific use cases that do not require a complex revision. Although evaluating automatic text revision is challenging, we conducted both human and automated evaluations of the revisions generated by the AI model. We found that most paragraphs were enhanced, while in some cases the model removed important information or introduced errors. The AI model also highlighted certain paragraphs that were difficult to revise, which could pose challenges for human readers as well.

Our approach has some limitations. We found that revising abstracts proved more challenging for the model, as revisions often removed background information about the research problem. There are opportunities to improve the AI-based revisions, such as further refining prompts using few-shot learning,^14^ or fine-tuning the model using an additional corpus of academic writing focused on particularly challenging sections. Fine-tuning using preprint-publication pairs^15^ may help to identify sections or phrases likely to be changed during peer review. Our approach processed each paragraph of the text but lacked a contextual thread between queries, which mainly affected the “Results” and “Methods” sections. Using chatbots that retain context could enable the revision of individual paragraphs while considering previously processed text. We plan to update our workflow to support this strategy. Regarding the LLM used, open and semi-open models, such as BLOOM,^16^ Meta’s Llama 2,^17^ and Mistral 7B,^18^ are growing in popularity and capabilities, but they lack the user-friendly OpenAI API. We used the LLM-as-a-Judge method to automatically assess the quality of revisions, which has limitations such as the self-enhancement bias where LLMs tend to favor text generated by themselves. Although our approach is based on revising human-generated text (rather than generating answers from scratch), we used 2 LLM judges, GPT-3.5 and GPT-4, to address this potential issue. These 2 models have shown limited self-enhancement bias and high alignment with human preferences.^13^ In this study, we found that the automated assessments were consistent with our human evaluations. Despite these limitations, we found that models captured the main ideas and generated a revision that often communicated the intended meaning more clearly and concisely. While our study focused on OpenAI’s GPT-3 and GPT-3.5 Turbo for revisions, the Manubot AI Editor is prepared to support future models.

## Conclusions

The use of AI-assisted tools for scientific authoring is controversial.^19^^,^^20^ Questions arise concerning the originality and ownership of texts generated by these models. For example, the *Nature* journal has established that any use of these models in scientific writing must be documented,^21^ and the International Conference on Machine Learning has prohibited the submission of “papers that include text generated from a large-scale language model (LLM),”^22^ although editing tools for grammar and spelling correction are allowed. Our work, however, focuses on revising *existing* text written by a human author. Additionally, all changes made by humans and AI are tracked in the version control system, which allows for full transparency. Despite the concerns, there are also significant opportunities. Our work lays the foundation for a future in which humans and machines construct academic manuscripts together. Scientific articles need to adhere to a certain style, which can make the writing time-consuming and require a significant amount of effort to think about *how* to communicate a result or finding that has already been obtained. As machines become increasingly capable of improving scholarly text, humans can focus more on *what* to communicate to others, rather than on *how* to write it. This could lead to a more equitable and productive future for research, where scientists are only limited by their ideas and ability to conduct experiments to uncover the underlying organizing principles of ourselves and our environment.

## Supplementary Material

ocae139_Supplementary_Data

## Data Availability

The manuscripts’ text and PRs used for human assessment are available on their GitHub repositories, at https://github.com/greenelab/ccc-manuscript, https://github.com/greenelab/phenoplier_manuscript and https://github.com/greenelab/manubot-gpt-manuscript. The manuscripts’ text and PRs used for the automated assessment are available at https://github.com/pivlab/manubot-ai-editor-code-test-mutator-epistasis-manuscript, https://github.com/pivlab/manubot-ai-editor-code-test-biochatter-manuscript, https://github.com/pivlab/manubot-ai-editor-code-test-phenoplier-manuscript, and https://github.com/pivlab/manubot-ai-editor-code-test-ccc-manuscript. The source code of the Manubot AI Editor is available at https://github.com/manubot/manubot-ai-editor and the code for the automated assessment of paragraph quality is available at https://github.com/pivlab/manubot-ai-editor-code. A DOI-citable version of this manuscript is available at https://doi.org/10.1101/2023.01.21.525030
