# Supplementary material for: A publishing infrastructure for Artificial Intelligence (AI)-assisted academic authoring
Source: J Am Med Inform Assoc. 2024 Jun 14;31(9):2103–13. doi: 10.1093/jamia/ocae139 (PMC11339502; doi:10.1093/jamia/ocae139)
Supplement: ocae139_Supplementary_Data [file ocae139_supplementary_data.zip › ocae139_Supplementary_Data/Supplementary Material - Manubot AI Editor - v04.0.pdf]

Supplementary Material

Installation and use

The Manubot AI Editor is part of the standard Manubot template manuscript, referred to as rootstock, and is available at https://github.com/manubot/rootstock. Users wishing to use the workflow only need to follow the standard procedures to install Manubot. The section "AI-assisted authoring," found in the file `USAGE.md` of the rootstock repository, explains how to enable the tool. Afterward, the workflow (named `ai-revision`) will be available and ready to use under the Actions tab of the user's manuscript repository.

Implementation details

To run the workflow, the user must specify the branch that will be revised, select the files/sections of the manuscript (optional), specify the language model to use, provide an optional custom prompt (section-specific prompts are used by default), and provide the output branch name. For more advanced users, it is also possible to modify most of the tool's behavior or the language model parameters.

When the workflow is triggered, it downloads the manuscript by cloning the specified branch. It revises all of the manuscript files, or only some of them if the user specifies a subset. Next, each paragraph in the file is read and submitted to the OpenAI API for revision. If the request is successful, the tool will write the revised paragraph in place of the original one, using one sentence per line (which is the recommended format for the input text). If the request fails, the tool might try again (up to five times by default) if it is a common error (such as "server overloaded") or a model-specific error that requires changing some of its parameters. If the error cannot be handled or the maximum number of retries is reached, the original paragraph is written instead, with an HTML comment at the top explaining the cause of the error. This allows the user to debug the problem and attempt to fix it if desired.

As shown in Figure 1b, each API request comprises a prompt (the instructions given to the model) and the paragraph to be revised. Unless the user specifies a custom prompt, the tool will use a section-specific prompt generator that incorporates the manuscript title and keywords. Therefore, both must be accurate to obtain the best revision outcomes. The other key component to process a paragraph is its section. For instance, the abstract is a set of sentences with no citations, whereas a paragraph from the Introduction section has several references to other scientific papers. A paragraph in the Results section has fewer citations but many references to figures or tables and must provide enough details about the experiments to understand and interpret the outcomes. The Methods section is more dependent on the type of paper, but in general, it has to provide technical details and sometimes mathematical formulas and equations. Therefore, we designed section-specific prompts, which we found led to the most useful suggestions. Figure and table captions, as well as paragraphs that contain only one or two sentences and fewer than sixty words, are not processed and are copied directly to the output file.

The section of a paragraph is automatically inferred from the file name using a simple strategy, such as if "introduction" or "methods" is part of the file name. If the tool fails to infer a section from the file, the user can still specify to which section the file belongs. The section can be a standard one (abstract, introduction, results, methods, or discussion) for which a specific prompt is used (Figure 1b), or a non-standard one for which a default prompt is used to instruct the model to perform basic revision. This includes *"minimizing the use of jargon, ensuring text grammar is correct, fixing spelling errors, and making sure the text has a clear sentence structure."*

Properties of language models

The Manubot AI Editor uses the Chat Completions API to process each paragraph. We have tested our tool using the Davinci (`text-davinci-003`, based on the initial GPT-3 models) and GPT-3.5 Turbo models (`gpt-3.5-turbo`). All models can be adjusted using different parameters (refer to OpenAI - API Reference), and the most important ones can be easily adjusted using our tool.

Language models for text completion have a context length that indicates the limit of tokens they can process (tokens are common character sequences in text). This limit includes the size of the prompt and the paragraph, as well as the maximum number of tokens to generate for the completion (parameter `max_tokens`). To ensure we never exceed this context length, our AI-assisted revision software processes each paragraph of the manuscript with section-specific prompts, as shown in Figure 1b. This approach allows us to process large manuscripts by breaking them into smaller chunks of text. However, since the language model only processes a single paragraph from a section, it can potentially lose the context needed to produce a better

output. Nonetheless, we find that the model still produces high-quality revisions (see Results). Additionally, the maximum number of tokens (parameter `max_tokens`) is twice the estimated number of tokens in the paragraph (one token approximately represents four characters, see OpenAI - Tokenizer). The tool automatically adjusts this parameter and performs the request again if a related error is returned by the API. The user can also force the tool to either use a fixed value for `max_tokens` for all paragraphs or change the fraction of maximum tokens based on the estimated paragraph size (two by default).

The language models used are stochastic, meaning they generate a different revision for the same input paragraph each time. This behavior can be adjusted by using the "sampling temperature" or "nucleus sampling" parameters (we use `temperature=0.5` by default). Although we selected default values that work well across multiple manuscripts, these parameters can be changed to make the model more deterministic. The user can also instruct the model to generate several completions and select the one with the highest log probability per token, which can improve the quality of the revision. Our implementation generates only one completion (parameter `best_of=1`) to avoid potentially high costs for the user. Additionally, our workflow allows the user to process either the entire manuscript or individual sections. This provides more cost-effective control while focusing on a single piece of text, wherein the user can run the tool several times and pick the preferred revised text.

Prompt engineering

We extensively tested our tool, including prompts, using a unit testing framework. Our unit tests cover the general processing of the manuscript content (such as splitting by paragraphs), the generation of custom prompts using the manuscript metadata, and writing back the text suggestions (ensuring that the original style is preserved as much as possible to minimize the number of changes). More importantly, they also cover some basic quality measures of the revised text. This latter set of unit tests was used during our prompt engineering work, and they ensure that section-specific prompts yield revisions with a minimum set of quality measures. For instance, we wrote unit tests to check that revised Abstracts consist of a single paragraph, start with a capital letter, end with a period, and that no citations to other articles are included. For the Introduction section, we check that a certain percentage of citations are kept, which also attempts to give the model some flexibility to remove text deemed unnecessary. We found that adding the instruction *"most of the citations to other academic papers are kept"* to the prompt was enough to achieve this with the most capable model. We also wrote unit tests to ensure the models returned citations in the correct Manubot/Markdown format (e.g., `[@doi:...]` or `[@arxiv:...]`), and found that no changes to the prompt were needed for this (i.e., the model automatically detected the correct format in most cases). For the Results section, we included tests with short inline formulas in LaTeX (e.g., `$\gamma_l$`) and references to figures, tables, equations, or other sections (e.g., `Figure @id` or `Equation (@id)`) and found that, in the majority of cases, the most capable model was able to correctly keep them with the right format. For the Methods section, in addition to the aforementioned tests, we also evaluated the ability of models to use the correct format for the definition of numbered, multiline equations, and found that the most capable model succeeded in most cases. For this particular case, we needed to modify our prompt to explicitly mention the correct format of multiline equations (see prompt for Methods in Figure 1).

We also included tests where the model is expected to fail in generating a revision (for instance, when the input paragraph is too long for the model's context length). In these cases, we ensure that the tool returns a proper error message. We ran our unit tests across all models under evaluation.
